# The Effects of a Five-Month Lockdown Due to COVID-19 on Physical Fitness Parameters in Adolescent Students: A Comparison between Cohorts

**DOI:** 10.3390/ijerph19010326

**Published:** 2021-12-29

**Authors:** Athanasios Tsoukos, Gregory C. Bogdanis

**Affiliations:** School of Physical Education and Sports Science, National and Kapodistrian University of Athens, 17237 Athens, Greece; gbogdanis@phed.uoa.gr

**Keywords:** pandemic, inactivity, upper body power, sprint, throwing, vertical jump, agility

## Abstract

Background: This study examined the effects of a five-month lockdown due to COVID-19 pandemic on physical fitness parameters in urban adolescent male and female students. Methods: Two hundred and ninety-three male and female students (age: 15.8 ± 0.3 years) who attended the fourth grade of the same high school during the years 2016–2017 (first control group), 2018–2019 (second control group) and 2020–2021 (lockdown group) took part in the present study. Results: The percentage of overweight and obese students, according to body mass index, increased in males from 16.0% (2016–2017) and 14.6% (2018–2019), to 36.7% in 2020–2021 (*p* < 0.01), and in females from 8.6% (2016–2017) and 7.0% (2016–2017), to 25.6% in 2020–2021 (*p* < 0.01). Lower body fitness, as assessed by jumping, sprinting and agility tests, was impaired for both males and females after the lockdown compared with the 2016–2017 and 2018–2019 cohorts (vertical jumps: 10.4–15.1%; *p* < 0.01; d = 0.58–1.01, 30 m sprint: 3.7–4.9%; *p* < 0.01; d = 0.62–0.74; 505 agility test: from 6.1% to 9.4%; *p* < 0.01; d = 0.80–1.04). However, flexibility and performance in upper-body fitness tests (handgrip maximum isometric strength and medicine ball throws with different loads) was significantly reduced only in males after the lockdown (*p* < 0.05 to 0.01). Conclusions: These results suggest that a five-month lockdown negative influenced the physical fitness of adolescent students. Notably, greater reductions were observed in upper body strength, power and flexibility in males than in females. These results highlight the need to maintain strength, power and body mass during long periods of inactivity in adolescent populations.

## 1. Introduction

Since December 2019, the 2019 coronavirus disease (COVID-19) has affected more than 250 million of people worldwide [1], and in March 2020 the World Health Organization (WHO) characterized COVID-19 as a pandemic [2]. To limit the spread of COVID-19 and protect human health and well-being, the governments of most countries imposed lockdown periods, during which people had to stay at home and were only allowed to go out for a limited time during the day. Lockdowns and similar types of restrictions in different countries lasted from some days to several months [3], and this increased the incidence of anxiety, depression, insomnia, and other psychological problems [4,5,6,7,8]. Furthermore, there was a dramatic decrease of physical activity (PA), especially in the younger population, which may decrease health-related quality of life [9] and may increase the likelihood of developing obesity as well as cardiovascular and metabolic diseases [10,11,12].

PA has been defined as any bodily movement produced by contraction of skeletal muscle or the total amount of time spent engaged in daily life activities that substantially increases energy expenditure [4,13,14]. Recent research has shown that, during COVID-19 lockdowns or other restrictions, PA decreases, especially in adolescents [15,16]. For example, Ruiz-Roso et al. [16] observed that PA status was significantly reduced in Latin American adolescents, mostly females, and this was accompanied by an increased frequency of consumption of ultra-processed foods. On the other hand, Sekulic et al. [15] evaluated changes in PA levels before and three weeks after the initiation of COVID-19 social distancing rules and observed a significant decrease of PA levels for the total sample which was primarily influenced by a significant decrease of PA levels in boys. Similarly, Elnaggar et al. [17] showed that PA was reduced during the COVID-19 pandemic restrictions and these changes were gender related, with adolescent boys presenting significant reductions whereas girls did not. The degree of PA decrease may be influenced by the living environment (country or continent, urban or rural) [14,16], educational, political and economic factors [18], and the prior level of physical fitness in adolescence [19,20].

Physical fitness includes various aspects of human performance such as speed, balance, agility, coordination, reaction time, muscular strength and power, cardiorespiratory endurance, flexibility and body composition [21]. A recent study showed that physical fitness parameters and anthropometric measurements of urban adolescents predicted their PA level during the COVID-19 pandemic [14]; however, the authors did not present any data from the physical fitness tests. Another study investigated the impact of the COVID-19 lockdown on fitness indices in adolescents and found a decrease of the number of sit-ups and 600 m time respectively, especially in boys, in the lockdown cohort compared with the control cohort [19]. However, these tests of local muscular endurance and aerobic/anaerobic capacity do not cover the entire spectrum of physical fitness components. To our knowledge, the few studies examining the effect of lockdowns or COVID-19 restrictions on physical fitness were performed in athletes [22,23,24,25], whereas a study in adolescent students showed only correlations of physical fitness tests with PA levels [15]. Taking into consideration the above and the sparsity of studies in adolescents, the purpose of the present study was to examine the effects of a 5-month lockdown during the year 2020–2021 on a wide range of fitness parameters (speed, agility, lower and upper body power, maximum isometric upper body strength, flexibility and body composition) in urban adolescent male and female students compared with their counterparts of the same age of the previous years: 2016–2017 and 2018–2019. A secondary purpose was to examine if males responded differently to reduced PA compared with females.

## 2. Materials and Methods

### 2.1. Subjects

Power analysis indicated a minimum sample size of 111 subjects per gender would be needed to detect a moderate effect size of 0.30. Power analysis was performed using the following parameters: Type of analysis was set to one-way between analysis of variance (ANOVA) groups, the required power was set to 0.80, alpha was set to 0.05, and the correlation between repeated measures was set to r = 0.5 (G-Power software, v.3.1.9.2).

Two hundred and ninety-three (n = 293) adolescent students (166 females, age: 15.8 ± 0.3 years, height: 1.63 ± 0.06 m, body mass: 57.0 ± 8.2 kg and 127 males, age: 15.9 ± 0.3 years, height: 1.75 ± 0.06 m, body mass: 67.7 ± 11.9 kg) took part in the study. We used the following inclusion criteria: (a) free of musculoskeletal injuries for at least 1 year prior to the study, (b) not taking any drugs or nutritional supplements, (c) non-smokers, (d) regular participation in physical education lessons (i.e., >80% of the total number of lessons).

The parents of the participants were informed in writing about the aim of the study and the possible risks involved and they signed an informed consent form. The study was approved by the institutional review board at the School of P.E. and Sport Science, National and Kapodistrian University of Athens (Approval no. 1282), and all procedures were in accordance with the Code of Ethics of the World Medical Association (Helsinki declaration of 1964, as revised in 2013).

### 2.2. Experimental Design

This is a cohort study comparing different groups of students (4th grade of high-school), who studied at the same school at three different school years (2016–2017, 2018–2019 and 2020–2021). These students followed the same physical education curriculum applied by the same physical education teacher (author A.T.). The data from the first two school years (2016–2017 and 2018–2019) were used as control measures, while the results of the students of 2020–2021 were used to examine the effects of a five-month pandemic lockdown due to the COVID-19 on physical fitness parameters.

Every April of each year, the same physical education teacher performed a standardized battery of fitness measurements in all 4th grade students at the school. We chose to analyze and compare the measurements performed in seasons 2016–2017, 2018–2019 and 2020–2021, because in 2019–2020 no measurements were performed because of the first unexpected lockdown and in the 2017–2018 season the dataset of measurements was not complete due to practical problems. All measurements were performed using the same sports facilities and on the same hour of the day (between 10–12 a.m.). The students were instructed to wear athletic clothes and shoes. The variables measured were: body height and mass, sum of four skinfolds (as a measure of adiposity), 30 m sprint time, 505 agility test, countermovement jump (CMJ), countermovement jump with free arms (CMJFA), maximum throwing velocity of balls with different weights, flexibility and hand-grip strength. These fitness tests were chosen primarily based on convenience at this school setting, and also because they assess most aspects of physical fitness (speed, agility, lower and upper body power, maximum strength, flexibility and body composition). Cardiorespiratory endurance was not assessed because it required a test to exhaustion, for which we did not receive permission from the students’ parents.

During the school years 2016–2017 and 2018–2019 the students followed the course of the first class of physical education from the middle of September until April whereas during the 2020–2021 school year the students attended the physical education classes from the middle of September until the 7th of November 2020. On the 7th of November of 2020, the Greek government announced a pandemic lockdown due to COVID-19 spread which lasted until the 11th of April 2021 (5 months). All measurements were conducted by one of the authors of the present study, A.T., who was the physical education teacher in the 2nd Experimental Lyceum of Athens (Greece) from 2013 until August of 2021.

### 2.3. Anthropometric Measurements

A stadiometer (Charder HM-200P Portstad, Charder Electronic Co Ltd., Taichung City, Taiwan) was used to measure body height to the nearest 0.01 cm and body mass was measured to the nearest 0.1 kg by a scale (TBF-300A Body Composition Analyzer-Tanita). Adiposity was assessed by the sum of 4 skinfold thicknesses (biceps, triceps, subscapular and suprailiac) using a Harpenden skinfold caliper (British Indicators Ltd., Herts, England). Body mass index (BMI) was calculated by dividing body mass by the square of body height. Percentiles for the BMI for age and gender, were calculated using the LMS method as previously described [26]. Individuals with BMI at or above the 85th percentile were considered as overweight/obese.

### 2.4. Familiarization and Standardized Warm-Up

Fitness tests were conducted 2–5 days apart during physical education classes between 10–12 a.m., following a standardized warm-up. The warm-up consisted of 5 min of light jogging on the court and 10 min of dynamic stretching of the lower and upper body muscles [27,28]. After that, participants performed a specific warm-up depending on the subsequent test, that included either short bouts of running and jumping drills, changes of direction or throwing movements or submaximal flexibility efforts or submaximal hand-grip efforts [27,28]. All students were thoroughly familiarized with all fitness tests during the previous physical education classes.

### 2.5. Vertical Jump Tests

Countermovement jump (CMJ) and countermovement jump with arm swing (CMJAS) were evaluated by an optical measurement system, which measured flight time (Optojump next, Microgate, Bolzano, Italy). During the CMJ, students were asked to jump as high as possible with their arms akimbo, while maintaining the same body position during take-off and landing [29]. The CMJAS was performed with the same technique as the countermovement jump but with an arm swing. Three jumps were performed in each type of the jumps with 30–45 s recovery between the jumps and the best performance was used for further analysis.

### 2.6. Sprint Test

The time to complete a 30 m sprint was measured using a telemetric timing system (Witty, Microgate, Italy) on two consecutive basketball courts. Cones and tape markers were placed on the ground at the start and at the end line. The students stood 30 cm behind the first set of photocells with a staggered stance [30] and the height of the photocells was 60 cm from the floor [30]. Each student performed two sprints with 3 min of recovery in between. The fastest performance was used for analysis.

### 2.7. Agility Test

For the 505 agility test, cone and tape markers were placed on the floor at the start and at 10 m and 15 m away from the starting line. A photogate (Witty, Microgate, Italy) was set laterally of the 10 m markers. The students ran as fast as possible from the starting line to the 15 m line, changed direction (180-degree turn) and ran as fast as possible returning to the 10 m line. The recorded time was the distance from the 10 m marker to the 15 m and back to the 10 m marker (a total of 10 m) [31]. Three trials were performed with 5 min rests in between, and the best result was kept for analysis.

### 2.8. Hand-Grip Strength

A hand-grip dynamometer (Takei Kiki Kogyo, Tokyo, Japan) was used to measure hand-grip strength. The students were seated on a chair with their torso upright, shoulders adducted, and the elbow flexed at 90 degrees [27,32]. They supported the dynamometer by positioning their hand on a table. They were instructed to squeeze as hard as possible for 5 s with a 1 min rest between each effort. Three trials for each hand (dominant and non-dominant) were performed, and the best performance was recorded. We calculated the hand-grip strength as the sum of the absolute maximum strength of both hands and the relative strength as the maximum strength of both hands divided by body mass.

### 2.9. Ball Throw Test

The ball throw test was used to assess the upper body power by throwing balls of different weights: a volleyball (mass = 0.270 kg), a 1 kg medicine ball, and a 3 kg medicine ball (MB3) in a randomized order. The maximum velocity of each medicine ball was measured using a Doppler radar gun (Sports Radar 3300; Sports Electronics, Inc., accuracy ± 0.028 m∙s^−1^). The radar gun was placed 1 m behind the student at ball height during the throw. The throws were performed in parallel with the ground, i.e., within a field of 10 degrees from the level of the gun [27]. The participants were instructed to throw the ball as fast as possible with both arms over their head, aiming at a square target on a wall which was at the same level as the ball and located 3 to 8 m away depending on the mass of the ball and the gender. The students stood with their feet parallel to each other and did not leave the ground during the throw. No preliminary steps were allowed before the throw [33]. Each subject performed 3 attempts with each ball with 2-min rest between each attempt. The order of the ball loads was randomized and balanced [27]. The highest speed for each ball weight was used for further analysis.

### 2.10. Flexibility Test

The sit-and-reach test was used to assess the flexibility of the hamstrings and lower back muscles of the participants [34]. The students took a seated position with legs fully extended and the feet (no shoes) were placed flat against a standardized sit-and-reach box. An investigator held the knees locked down and the subjects reached forward with both hands as far as possible, keeping the palms facing downwards and the hands on top of each other. The zero mark was 15 cm before the feet.

### 2.11. Statistical Analysis

Statistical analyses were carried out with SPSS (IBM SPSS Statistics Version 23). All data are presented as means and standard deviations (SD). One-way between-groups ANOVA was conducted to determine the differences between years (2017, 2019 and 2021) in males and females separately. A Tukey’s post hoc test was performed when a significant main effect or interaction was observed. The effect sizes were determined by Partial eta squared (η^2^) values. Partial eta squared (η^2^) values were classified as small (0.01 to 0.059), moderate (0.06 to 0.137) and large (>0.137). For pairwise comparisons, effect size was determined by Cohen’s d (small: 0.2, medium: 0.5, large: 0.8). Statistical significance was set at *p* ≤ 0.05.

## 3. Results

### 3.1. Anthropometric Measurements

The one-way ANOVA showed that age (males: *p* = 0.59, η^2^ = 0.009; females: *p* = 0.19, η^2^ = 0.02) and the body height (males: *p* = 0.79, η^2^ = 0.003; females: *p* = 0.17, η^2^ = 0.02) were not different between years in both males and females, respectively. In contrast, one-way ANOVA was significant in body mass in both males (*p* = 0.024, η^2^ = 0.06) and females (*p* = 0.001, η^2^ = 0.06). Tukey’s post hoc tests indicated that both male and female students had a significantly greater body mass in the 2020–21 year compared with their counterparts in 2016–2017 and 2018–2019, respectively (Table 1). No difference was observed between 2016–2017 and 2018–2019.

One-way ANOVA showed a similar finding (between-groups effect) for BMI (males: *p* < 0.001, η^2^ = 0.09; females: *p* < 0.001, η^2^ = 0.06). Tukey’s post hoc tests indicated that students had a significantly higher BMI in 2020–21 compared with their counterparts in 2016–2017 and 2018–2019 respectively (Table 1). No difference was observed between 2016–2017 and 2018–2019. Percentile rankings for BMI showed that in 2020–2021 the percentage of male and female participants characterized as overweight/obese was increased by 2 to 3 times, compared with the years 2016–2017 and 2018–2019 (Table 1).

One-way ANOVA was significant in the sum of skinfolds only in males (*p* = 0.019, η^2^ = 0.07). Tukey’s post hoc tests showed a significant difference between the year 2020–2021 compared with 2016–2017 and 2018–2019 respectively (Table 1). No difference in the sum of skinfolds was found for females (*p* = 0.60, η^2^ = 0.006).

### 3.2. Effects on Fitness Tests

#### 3.2.1. Effects on Lower Body Fitness Tests and Flexibility

The effects of the lockdown (2020–2021) on the lower body fitness tests followed a similar trend (decrease of performance) for males and females (Table 2). For CMJ and CMJAS, one-way ANOVA presented a significant between-groups effect for both males (CMJ: *p* = 0.013; η^2^ = 0.08, CMJAS: *p* = 0.010; η^2^ = 0.08) and females (CMJ: *p* < 0.001; η^2^ = 0.15, CMJAS: *p* < 0.001; η^2^ = 0.14). Tukey’s post hoc tests indicated that male and female students had a significantly lower CMJ and CMJ performance in 2020–2021 year compared with their counterparts in 2016–2017 and 2018–2019 (Table 2).

For the 505 agility test and 30m sprint, the one-way ANOVA also showed a significant between-groups effect for both males (505 agility: *p* < 0.0001; η^2^ = 0.20, 30 m sprint: *p* = 0.003; η^2^ = 0.10) and females (505 agility: *p* < 0.001; η^2^ = 0.16, 30 m sprint: *p* < 0.01; η^2^ = 0.09). Tukey’s post hoc tests indicated that all students had a significantly lower 505 agility test and 30 m sprint performance (longer times) in 2020–2021 year compared with their counterparts in 2016–2017 (Table 2).

For the sit-and-reach test, one-way ANOVA revealed significant differences for males (*p* = 0.015; η^2^ = 0.08) but not for females (*p* = 0.14; η^2^ = 0.03). Tukey’s post hoc tests revealed that the sit-and-reach test performance in 2020–2021 year was lower only in males by −23.6% (*p* < 0.05) and −24.4% (*p* < 0.05) and not in females, although there was a change of −10.1% and −10.5% from 2016–2017 and 2018–2019 respectively (Table 2).

#### 3.2.2. Effects on Upper Body Fitness Tests

**Figure 1:**
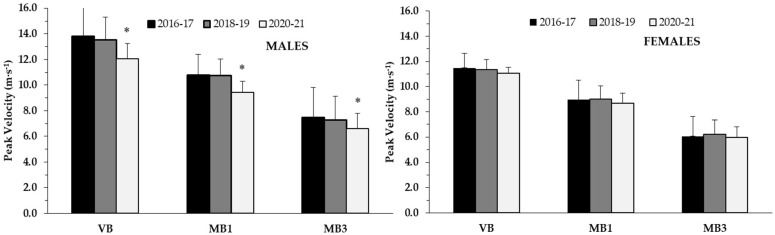
Ball throwing with different loads at different school years in males (left panel) and females (right panel) presented as means ± SD. *: *p* < 0.01 from 2016–2017 and 2018–2019.

Changes in upper body fitness test performance in 2020–2021 were different only in males but not in females. For the throwing tests with different ball weights, one-way ANOVAs were significant for males (volleyball throw: *p* < 0.01; η^2^ = 0.10, 1 kg MB throw: *p* < 0.01; η^2^ = 0.09 and 3 kg MB throw: *p* < 0.01; η^2^ = 0.10). Tukey’s post hoc tests indicated that males had a significantly lower throwing performance during 2020–2021 in all loads compared with their counterparts in 2016–2017 (volleyball throw: −12.3%; *p* < 0.01; d = 0.76, 1 kg MB throw: −13.0%; *p* < 0.01; d = 0.82, 3 kg MB throw: −12.0%; *p* < 0.01; d = 0.76) and 2018–2019 (volleyball throw: −10.4%; *p* < 0.01; d = 0.71, 1 kg MB throw: −13.0%; *p* < 0.01; d = 0.92, 3 kg MB throw: −9.6%; *p* < 0.01; d = 0.71), respectively (Figure 1). In contrast, one-way ANOVAs were not significantly different for females (volleyball throw: *p* = 0.43; η^2^ = 0.01, 1 kg MB throw: *p* = 0.24; η^2^ = 0.02 and 3 kg MB throw: *p* = 0.27; η^2^ = 0.02) (Figure 1).

The hand-grip strength (maximum isometric) and relative hand-grip strength measurements also revealed significant one-way ANOVAs for males (hand-grip strength: *p* = 0.035; η^2^ = 0.06, relative hand-grip strength: *p* < 0.01; η^2^ = 0.11) and no significant difference for females (hand-grip strength: *p* = 0.35; η^2^ = 0.01, relative hand-grip strength: *p* = 0.27; η^2^ = 0.02).

**Figure 2:**
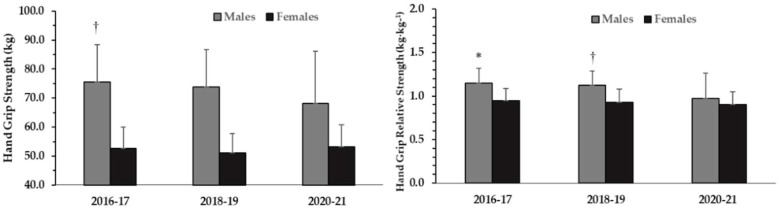
Hand-grip Strength in absolute (left panel) and relative hand-grip strength (right panel) at different school years in males and females presented as means ± SD. *: *p* < 0.01 from 2020–2021, †: *p* < 0.05 from 2020–2021.

Tukey’s post hoc tests indicated that males had a significantly lower hand-grip strength and relative hand-grip strength during 2020–2021 compared with their counterparts in 2016–2017 (68.1 kg ± 18.1 kg vs. 75.5 kg ± 12.8 kg; *p* < 0.05; d = 0.51, relative hand-grip strength: −9.1%; *p* < 0.01; d = 0.42). Tukey’s post hoc tests also showed that hand-grip strength was not different between 2020–2021 and 2018–2019 although this difference was almost 7.7% (68.1 kg ± 18.1 kg vs. 73.8 kg ± 13.0 kg; *p* = 0.28; d = 0.38) (Figure 2). However, relative hand-grip strength in males was different between 2020–2021 and 2018–2019 (0.97 ± 0.29 kg∙kg^−1^ vs. 1.12 ± 0.16 kg∙kg^−1^, *p* < 0.05, d = 0.41) (Figure 2).

## 4. Discussion

The main finding of the present study was that body mass and BMI, as well as lower body fitness tests measurements were negatively affected by the 5 months of the COVID-19 lockdown (year 2020–2021) compared with the previous classes in both males and females (2016–2017 and 2018–2019). Furthermore, the upper-body fitness tests (handgrip maximum isometric strength, relative strength and medicine ball throws with different loads) and flexibility showed that performance was significantly reduced in males after the COVID-19 lockdown (2020–2021) compared with the previous classes which served as controls (2016–2017 and 2018–2019), whereas we upper body performance was unaffected in females.

The finding that both males and females had higher body mass and BMI after the COVID-19 lockdown may be attributed to the reduced levels of PA and the change in nutritional habits [14,15,16,17]. In a similar population of Greek adolescents, research has shown that moderate and vigorous physical activity were lower than 50% of the physical activity recommended by WHO and well-being generally was poor [35]. In addition, Giustino et al. [4] estimated the levels of PA, expressed as energy expenditure (MET–minute/week), among physically active adults before and during the last seven days of the COVID-19 quarantine and observed a significant decrease of the total weekly energy expenditure. In a recent study [14] PA levels were evaluated in 823 adolescents during the COVID-19 pandemic. The authors found that the adolescents who lived in an urban environment, similar to the sample of the present study, decreased their PA levels by 13.8% compared with before the implementation of lockdown [14]. Furthermore, research has shown that during a lockdown in Greece, body weight was increased and this was associated with decreased physical activity in children and adolescents [36]. Similarly, in another study, a semi-lockdown increased BMI of 70.3% of volleyball players in Cameroon [22]. A recent systematic review and meta-analysis showed significant increases in body mass and BMI during the lockdown in school-age children and adolescents due to physical inactivity and sedentary behavior [20,37]. A study conducted in Spain during the COVID-19 confinement showed a reduction of weekly minutes of physical activity during the confinement by 51.6%, and an increase of daily hours of screen exposure, i.e., TV watching and game playing (+2.9 ± 2.1 h) in 860 male and female children aged between 3 and 16 years [38]. In addition, the COVID-19 pandemic restrictions have been shown to increase food intake and the consumption of unhealthy food choices promoting weight gain [39]. Thus, the increase of body mass and BMI after the COVID-19 lockdown in the present study may be attributed to the reduction of PA, which favors inactivity, unhealthy food intake and sedentary behavior.

In the present study we showed that lower body fitness tests significantly differed between the class that experienced COVID-19 lockdown (2020–2021) compared with the previous classes in both males and females (2016–2017 and 2018–2019). To our knowledge, the few studies examining the effect of COVID-19 restrictions and lockdowns on similar fitness tests as those used in the present study, were conducted on athletes [22,23,24,25]. Salazar et al. [25] evaluated the impact of training cessation due to COVID-19 home confinement on physical performance of adolescent basketball players (17.5 ± 1.3 years old) and found significant reductions in vertical jumping ability (squat jump: −8.5%, CMJFA: −8.3%), sprint performance (5 m: 3.1%, 10 m: 3.9%, 15 m: 4.7%) and agility performance (10.6 to 11.0%). In the present study we showed greater decreases in vertical jumping ability (−13.6% to −15%) and similar impairments in sprint and agility performance (4.7% to 5.5% for 30 m sprint time and 7.9% to 8.3% on the 505 agility test) compared with the study of Salazar et al. [25]. These decrements in speed and power performance in adolescents could be attributed to reductions in muscle fiber cross-sectional area (muscular factor) and to an impaired central activation (neural factor) as a result of inactivity [40], which has been shown to decrease force production and electromyographic activity [41]. On the other hand, Spyrou et al. [23] did not observe significant differences in vertical jump after 70 days of quarantine in futsal elite players. These discrepancies in vertical jumping ability in students compared with athletes may be that athletes even in the case of lockdowns and quarantines were obliged to train at home [42]. In the present study we also observed that flexibility of lower back and hamstrings, measured by the sit-and-reach test, was significantly lower only in males after the 5-month lockdown compared with the previous cohorts. Similar reductions have been shown in professional male soccer players after a COVID-19 confinement period [43] which was accompanied by a decrease in hamstring eccentric strength for both the dominant and the non-dominant leg. The reductions in lower body fitness tests in the present study might be also attributed to the lower levels of PA which has been shown to be related with fitness tests [15]. Sekulic et al. [15] evaluated changes in PA levels in adolescents and found that performance in fitness tests (e.g., jumping capacity, abdominal strength, aerobic endurance and anaerobic endurance) was positively correlated with PA levels at baseline and follow-up among boys and girls. Similarly, Zenic et al. [14] revealed that the fitness status of urban adolescent students who were living in a similar environment and had a similar age to the students of the present study predicted their PA levels at follow-up, i.e., after the COVID-19 pandemic and social distancing implementation.

Another finding of the present study was that males had significantly lower upper-body performance after the COVID-19 lockdown compared with the previous classes who served as controls, while this reduction in strength and power was not observed in females. Sunda et al. [19] reported a greater impairment in 600 m run and sit-up performance in boys than in girls after two months of lockdown for lower body and torso performance [19]. One possible explanation may be the possibly greater decrease in PA in boys than in girls, as reported in two recent studies examining the influence of COVID-19 restrictions on PA levels in Croatia [15] and in Saudi Arabia [17]. The authors justified these results by the fact that PA levels among boys were mostly related to participation in organized sports because male adolescents engage more frequently in competitive sport than females [15,17]. Indeed, Marques et al. [44] examined whether participation in organized sports is related to achieving physical activity recommendations, body mass index (BMI), objectively measured PA intensity and time spent sedentary in 973 children and adolescents and revealed that more boys (51.3%) than girls (28.3%) reported to be involved in organized sports participation. These results and the fact that most male students (25% more compared with girls) in Greece participated in soccer and basketball, volleyball, athletics, swimming, handball, Tae-Kwon-Do and gymnastics [45], in which the use of upper body strength is vital, may explain the results of the present study. Thus, the implementation of the COVID-19 lockdown and the banning of organized sports participation may have resulted in a greater decrease in PA, especially of the upper body, in males more so than in females in the present study. In addition, the greater upper body strength and muscle mass in boys compared with girls at the age of 16 years may render them more susceptible to loss of strength and power during periods of inactivity [46].

The present study has some limitations which should be mentioned. We did not collect any qualitative data, such as the participation of the students in sports training outside the school environment and the level of habitual PA. In addition, possible differences in the ideal images of body fitness and lifestyles promoted by the media across time in the three different cohorts may have also affected the results. Another limitation is that it was not possible to conduct a pre- vs. post-lockdown study, since the lockdown was unexpected. Finally, qualitative data, such as lifestyle, dietary habits, sleep duration and physiological parameters would be useful to get a complete picture of the influence of a lockdown on fitness, health, and welfare of adolescents.

## 5. Conclusions

In conclusion, the present study showed that body mass and BMI was higher, and performance on several fitness tests was lower in the class that experienced COVID-19 lockdown (2020–2021) compared with the previous classes in both males and females (2016–2017 and 2018–2019). Importantly, while changes in lower body performance were similar in males and females, upper-body strength and power was lower only in males after the COVID-19 lockdown (2020–2021), compared with students of previous years who served as controls (2016–2017 and 2018–2019). As speed, power and agility of the lower limbs is equally affected in male and female adolescent students after a 5-month lockdown, attention should be given to maintain leg power during a long period of restricted physical activity. In addition, maintaining body weight is important, since relative strength and power (i.e., scaled by body mass) influences speed, agility and jump performance. Finally, the decrease of upper body strength and flexibility only in boys suggests that there is a gender difference in the effects of lockdown in adolescent populations.

## Figures and Tables

**Table 1:** Anthropometric measurements at different school years by sex (mean ± SD).

Males					
Variables	2016–2017 N = 52	2018–2019 N = 42	2020–2021 N = 30	Cohen’s d (2016–2017 to 2020–2021)	Cohen’s d (2018–2019 to 2020–2021)
Age	15.9 ± 0.3	15.8 ± 0.2	15.9 ± 0.3	0.00	0.41
Height (m)	1.75 ± 0.06	1.75 ± 0.07	1.75 ± 0.05	0.00	0.00
Body Mass (kg)	66.1 ± 8.6 *	65.8 ± 10.4 *	72.8 ± 16.6	0.56	0.53
BMI (kg∙m^2^)	21.7 ± 2.5 *	21.3 ± 2.6 **	23.8 ± 4.9	0.60	0.69
Skinfolds (mm)	36.6 ± 14.5 *	36.5 ± 14.2 *	47.8 ± 26.8	0.57	0.56
Overweight/obese (% of total)	16.0%	14.6%	36.7%		
**Females**					
**Variables**	**2016–2017 N = 60**	**2018–2019** **N = 61**	**2020–2021 N = 45**	**Cohen’s d (2016–2017 to 2020–2021)**	**Cohen’s d (2018–2019 to 2020–2021)**
Age	15.8 ± 0.3	15.8 ± 0.3	15.9 ± 0.3	0.34	0.34
Height (m)	1.62 ± 0.06	1.64 ± 0.05	1.64 ± 0.06	0.34	0.00
Body Mass (kg)	55.4 ± 6.4 **	56.0 ± 7.1 *	60.3 ± 10.5	0.59	0.50
BMI (kg∙m^2^)	21.2 ± 2.4 *	20.8 ± 2.3 **	22.5 ± 3.6	0.44	0.59
Skinfolds (mm)	55.8 ± 16.6	52.5 ± 17.1	55.5 ± 23.4	0.02	0.15
Overweight/obese (% of total)	8.6%	7.0%	25.6%		

**: *p* < 0.01 from 2020–2021; *: *p* < 0.05 from 2020–2021.

**Table 2:** Lower-body fitness test results at different school years by sex (mean ± SD).

Males					
Variables	2016–2017 (N = 44)	2018–2019 (N = 39)	2020–2021 (N = 41)	Cohen’s d (2016–2017 to 2020–2021)	Cohen’s d (2018–2019 to 2020–2021)
CMJ (cm)	32.8 ± 5.7 *	33.7 ± 5.9 *	29.4 ± 6.4	0.58	0.71
CMJ AS (cm)	38.4 ± 6.8 *	38.7 ± 6.9 *	34.1 ± 6.6	0.65	0.69
Flexibility (cm)	20.7 ± 7.7 *	20.9 ± 9.7 *	15.8 ± 13.3	0.49	0.44
505 agility (s)	2.65 ± 0.21 **	2.67 ± 0.17 **	2.90 ± 0.28	1.04	1.03
30 m sprint (s)	4.67 ± 0.27 **	4.69 ± 0.27 **	4.90 ± 0.39	0.70	0.63
**Females**					
**Variables**	**2016–2017 (N = 51)**	**2018–2019 (N = 39)**	**2020–2021 (N = 41)**	**Cohen’s d (2016–2017 to 2020–2021)**	**Cohen’s d (2018–2019 to 2020–2021)**
CMJ (cm)	23.9 ± 4.0 **	24.5 ± 4.0 **	20.8 ± 3.4	0.84	1.01
CMJ AS (cm)	27.2 ± 5.0 **	27.3 ± 4.6 **	23.3 ± 3.7	0.88	0.97
Flexibility (cm)	28.6 ± 6.9	28.7 ± 9.0	25.7 ± 8.5	0.39	0.35
505 agility (s)	2.90 ± 0.20 **	2.93 ± 0.19 **	3.11 ± 0.26	0.93	0.80
30 m sprint (s)	5.35 ± 0.30 *	5.30 ± 0.33 **	5.55 ± 0.36	0.62	0.74

**: *p* < 0.01 from 2020–2021; *: *p* < 0.05 from 2020–2021.

## Data Availability

The data are available upon request to the corresponding author.
